# Numeric Analysis on Shear Behavior of High-Strength Concrete Single-Keyed Dry Joints with Fixing Imperfections in Precast Concrete Segmental Bridges

**DOI:** 10.3390/ma13132914

**Published:** 2020-06-29

**Authors:** Haibo Jiang, Mingzhu Chen, Zhijun Sha, Jie Xiao, Jiahui Feng

**Affiliations:** Guangzhou Higher Education Mega Center, School of Civil and Transportation Engineering, Guangdong University of Technology, Guangzhou 510006, China; hbjiang@gdut.edu.cn (H.J.); mzchen@mail2.gdut.edu.cn (M.C.); zwh565979660@gmail.com (Z.S.); jhfeng@mail2.gdut.edu.cn (J.F.)

**Keywords:** precast concrete segmental bridges, single-keyed dry joints, high-strength concrete, finite-element model, shear strength, fixing imperfections of key

## Abstract

Fixing imperfections in keyed dry joints between the concrete segments compromise the performance of precast concrete segmental bridges (PCSBs), which needs to consider carefully. In this study, a finite-element model on high-strength concrete single-keyed dry joints in PCSBs was established and validated by experimental results. Parametric studies on fixing imperfections in key, concrete strengths, and confining pressures were carried out based on that model. The numeric results included crack patterns, load–displacements and shear strength. Fixing imperfections—especially at lower surface of keys—reduced shear strength of single-keyed dry joints by the different shear transfer mechanism. Higher confining pressure and concrete strength improved the shear strength, but they mitigated and aggravated the effect of fixing imperfections at lower surface of key on shear strength, respectively. Compared with simulating results, AASHTO standard overestimated the shear capacity of single-keyed dry joints with fixing imperfections at lower surface of key by up to 0.602–22.0%, but greatly underestimated that of the rest. A modified formula with a strength reduction factor was proposed. For six experimental three-keyed dry-joint specimens and 30 numeric single-keyed dry-joint specimens with or without fixing imperfections, the average ratio of code predictions to experimental results was 90.4% and 81.6%, respectively.

## 1. Introduction

**Figure 1:**
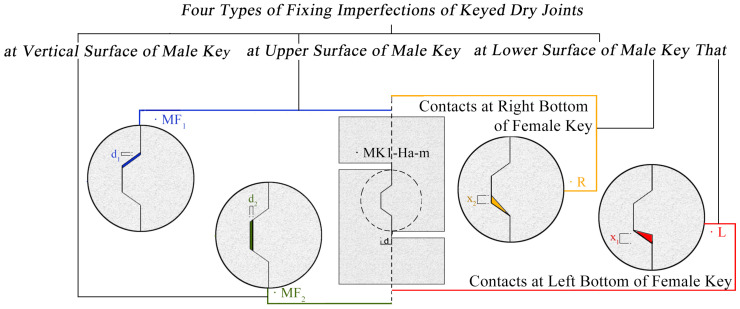
Fixing imperfections of keyed dry joints.

As concrete is the most wildly used construction material, more advanced-performance concrete structures, types of concrete, and construction methods are proposed and investigated [1,2,3,4]. Precast concrete segmental bridges (PCSBs) are widely constructed by bridge builders around the world for its economic, technical and social benefits [1,4,5,6]. However, the behavior and safety of PCSBs are affected by the joints between precast segments [1,6,7,8]. These joints, representing locations of discontinuity in the bridge, play a vital role in transmitting the compressive, shear stress and the deformation between the precast segments [4,7,8,9,10,11,12]. Nowadays, joints in PCSBs are commonly epoxied with a thin layer of epoxy [13,14,15,16]. Nevertheless, it was pointed out that the use of epoxy should be avoided if possible [17] to avoid the problem of smear quality of epoxy and brittle failure [15,16]. Dry joints are further studied as demand for fast construction and they significantly improve efficiency [18], economic, quality and security of construction in PCSBs [7,11,19,20,21,22,23]. Keyed dry joints are much often proposed than flat dry joints. Dry jointed segmental bridges mainly sustain shear stress between the segments [10] and three functions of shear keys are provided to enhance shear behavior of these joints [6,24]: connect the segments and align them, transfer the shear loads and permit durability as shear keys protect the tendons passing through the joints from corrosion. Using multiple castellated small keys in the key zone instead of single-keyed joints is the current trend to provide a better mechanical interlock [11,23,24]. However, stiffness and shear strength of the unreinforced keys are lower than those of adjacent monolithic sections within the segments [10], not only reducing the shear strength and stiffness, but changing the failure mode of the whole structure comparing to the monolithically cast-in-place beams [20,25,26,27]. In addition, these dry jointed shear keys have a higher possibility of fixing imperfections due to fabrication error or tiny deficiency from cracking (Figure 1).

Factors affecting shear behavior of keyed joints have been explored in several studies, including prestress levels, the shape of the key, surface preparation, concrete strength, contact area, friction coefficient and mechanical interlock of shear keys between segments [1,7,10,11,12,14,16,20,28,29,30]. Experimental studies on shear behavior of keyed dry joints under direct-shear load held by Buyukozturk et al. (1990) [11] and Zhou et al. (2005) [10] suggested two conclusions, also recorded in AASHTO [24]. One was the shearing-off failure mode (i.e., initiated by diagonal cracks at the right bottom of the corbel-like keys and propagated upward at approximately 45 degrees to the horizontal and then occurred when the keys were sheared off along the joint plane, representing the ultimate shear-off failure) influenced by factors above and further studied by Turmo, J. (2006b) [17], Jiang et al. (2016) [31], etc. In addition, these studies pointed out that the shear capacity of keyed dry joints basically consists of two parts [10,11,30,31], the shear friction force relative to confining stress levels between the flat surfaces and the shear contribution of keys relative to both the concrete strength and the confining stress levels. One of the most important factors is the concrete strength of joints, therefore, a series of studies on high-strength concrete (HSC) joints were presented to improve the shear behavior of joints [1,4,31]. Whereas the typical shear flow mechanisms across dry joints are studied extensively, there is still no consensus regarding their quantification. The typical procedures for the design of the keyed dry joints use an empirical relationship in the form of a simple analytical formula, such as the formula proposed by AASHTO [24], to prevent initial diagonal cracking and to prevent shear keys sheared off along the joint plane in PCSBs.

Though the formula from AASHTO is widely used to predict shear capacity of keyed dry joints in practical engineering, it is not always practical. According to several experimental studies [10,15,23,30,31,32,33], AASHTO underestimated the shear capacity of single-keyed dry joints while it always greatly overestimated that of multiple-keyed dry joints, which was also found in a series of numeric study by Rombach et al. (2004) [34], Turmo et al. (2012) [35], Alcalde et al. (2013) [36], Shamass et al. (2015) [37] and Chen et al. (2019) [14]. Moreover, for a shear key in keyed dry joints, the fact that shearing-off failure mode always occurs remains to be proved. A series of experimental tests about single-keyed and three-keyed joints [7,10,11,20,28,29] was conducted and found the crack patterns of shearing-off failure relative to AASHTO provision [24]. Several relative numeric studies from references [34,35,36,37,38] also underlined the shearing-off mode of keyed dry joints, single and multiple. However, Jiang et al. (2015) [39] proposed two modes of crack patterns from experimental results of keyed dry joints, single and multiple. Most of the crack patterns were mode one that the first crack formed at the bottom corner of the key and propagated 45 degrees in the horizontal direction before the second crack started to appear at the bottom of the key and developed toward the upper corner at an inclined angle of approximately 50–70° with respect to the horizontal line, instead of those occurred at the root of keys in single-keyed dry joints (shearing-off failure mode), that finally sheared off the keys. Then, Jiang et al. (2016) [31] found the mode one from the tests of three-keyed dry joints again. Similarly, Liu et al. (2018) [23] proposed the same crack propagations in three-keyed dry joints and advised that stress distributions in the shear plane need to be investigated in future research. Turmo et al. (2006a) [30] carried out an experimental study on shear behavior of seven-keyed dry joints reserved 3-mm separation between the faces of joints throughout the entire test and found that the shear keys progressively cracked in both male and female parts until the joints failed severely and suddenly under direct shear loads, damaged with the flat contact surface failed. Besides, Ahmed et al. (2019) [15] found that there were visible cracks in female keys of multiple-keyed dry joints which was an arc shaped path through both male and female keys. This problem was first presented by Zhou et al. (2005) [10] early before who conducted a series of experimental tests on joints, single-keyed and multiple-keyed, dry and epoxy, and speculated that the normalized shear stress of keys in three-keyed dry joints was lower than those in single-keyed dry joints and predicted from AASHTO specification due to the higher possibility of fixing imperfections of keys (Figure 1).

Surprisingly, the effects of fixing imperfections have not been closely and systematically studied due to limited experimental techniques and numeric discoveries until now. Zhou et al. (2005) [10], concluded that higher possibility of fixing imperfections in multiple-keyed dry joints led the load not to be uniformly distributed to each key to lead stress concentration to increase with different levels and cause the sequence failure of keys from bottom to top, the undeveloped overall shear capacity of keys and lower average ultimate shear strength of each key. Later Jiang et al. (2016) presented the same sequential failure of three-keyed dry joints, but no specific explanation [31]. That was confirmed by the contrast tests of epoxied joints from Zhou et al. (2005) [10] as epoxy alleviated the fixing imperfections in multiple-keyed joints and enabled the normalized shear stress per key in multiple-keyed joints comparable with single-keyed joints. A recent study carried by Ahmed et al. (2019) also pointed out that epoxied joints could provide a uniform load distribution than the dry [15]. Hence, a strength reduction factor should be introduced to AASHTO formula when applied in multiple-keyed dry joints. Ahmed et al. (2019) [15] proposed a reduction of AASHTO provision for multiple-keyed dry joints as 0.78 based on tests. Experimental results from Turmo et al. (2006a) [30] mentioned above also indicated how the fixing imperfections of keys were unfavorable to the shear capacity of keyed dry joints. Generally, fixing imperfections that were always possibly present in keyed dry joints may be key to the shear behavior of keyed dry joints, particularly the multiple-keyed.

As for the numeric study, there is no previous research on the effect of fixing imperfections in keyed dry joints. Rombach et al. (2004) [34], Turmo et al. (2012) [35], Alcalde et al. (2013) [36] and Shamass et al. (2015) [37] presented finite-element (FE) models of keyed dry joints without simulated fixing imperfections, which all indicated a conclusion that normalized shear stress of keys in multiple-keyed dry joints was lower even without fixing imperfections of keys and showed the typical shearing-off failure mode of each single shear key [38]. Rombach et al. (2004) [34] stated the simultaneous shear failure of keys in multiple-keyed dry joints and the lower overall shear capacity than AASHTO relationships [24]. Zou et al. (2011) [40] found out in a finite element model of five-keyed dry joints that the stress of each key was still not uniform under the same load and the stress distribution and displacement were both larger in the upper and lower keys and smaller in the middle part, among which the uppermost part was the largest. These numeric studies all diverged from the conclusion of the effect of fixing imperfections from Zhou et al. (2005) [10] and this means the summary provided by Zhou et al. (2005) [10] need to be systematically studied more as they were speculated from tests results. As a common, essential and controversial problem, it’s necessary to investigate the effect of fixing imperfections on shear behavior of keyed dry joints, including how they affect the stress distributions in the shear plane, the crack patterns—and decrease of shear capacity.

Since the parameters of fixing imperfections are hard to control in experimental tests and no previous investigation was systematically and consistently presented, this study tried to propose it by a numeric model. In this study, a numeric analysis, using a FE model established in ABAQUS [41], was conducted to investigate how the fixing imperfections of key effected on shear behavior of HSC single-keyed dry joints in PCSBs. First, the FE model was validated by the experimental data published by the authors [31], and then used for the parametric studies in terms of four fixing imperfections of key, confining stress levels and concrete strength. Crack patterns, the contour plat of the maximum principal stress contour, load–displacements, and ultimate shear loads were illustrated according to the numeric results. Lastly, the shear capacity simulated in these parametric studies were compared with those calculation based on the AASHTO formula [24]. Several interesting findings were revealed and a strength reduction factor as a modification for the AASHTO relationship to better predict shear capacity of keyed dry joints was presented by this study.

## 2. Materials and Methods

### 2.1. Concrete Damage Plasticity Model

**Figure 2:**
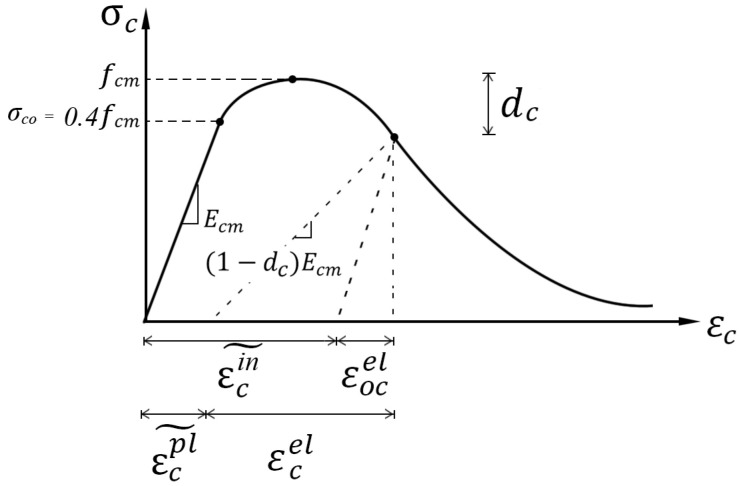
Concrete-damaged plasticity model in compression.

**Figure 3:**
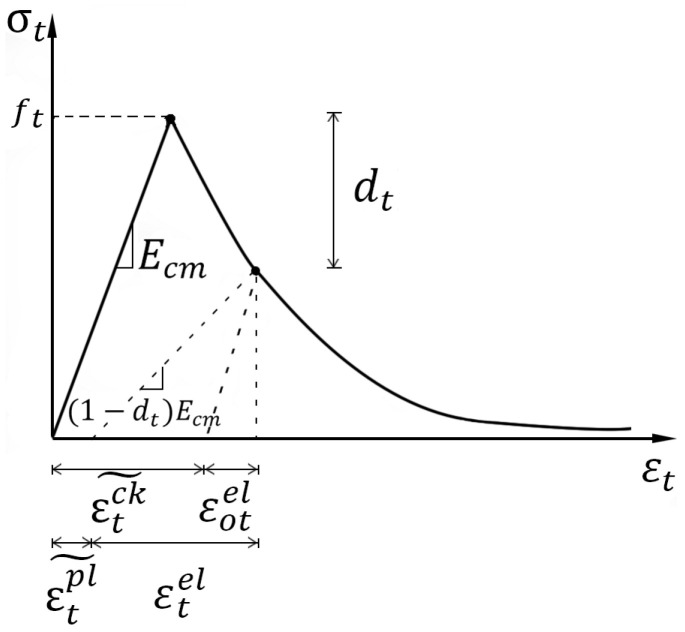
Concrete-damaged plasticity model in tension.

The concrete-damaged plasticity (CDP) model in *ABAQUS* code that supports the definition of inelastic behavior of concrete in compression and tension, including the tensile cracking failure and compressive crushing failure, was taken in this study for simulating damage in concrete in *ABAQUS 6.14* [41] (Figure 2 and Figure 3). The general CDP model parameters were chosen as below. Dilation angle, flow potential eccentricity, and viscosity parameter were set as 36, 0.1 and 0.001, respectively. The ratio of the strength in the biaxial state to the strength in the uniaxial state, $f_{b0}/f_{c0}$, was 1.16. The ratio of the second stress invariant on the tensile meridian, $K_{c}$, was 0.6667.

### 2.2. Concrete Constitutive Relation under Uniaxial Compression

To use the CDP model mentioned above for simulating concrete cracking and crack evolution history, stress–strain relationships in compression and in tension for concrete were required. In this study, the complete $\sigma_{k}-\varepsilon_{k}(k=c,t)$ curve proposed by Chinese code for the design of concrete structures (Specification for Design of Concrete Structure (GB 50010-2010)) [42] was used for concrete under compression and tension, which was suggested in the following illustration.

The modulus of elasticity $E_{c}$ and the poisson ratio $V_{c}$ of concrete are determined by the following equation:

(1) $$E_{c}=\frac{10^{5}}{2.2+\frac{34.7}{f_{cm}}}$$

(2) $$V_{c}=0.2$$

where $f_{cm}$ = average value of ultimate compressive strength of cube.

The constitutive relationship of concrete under uniaxial compression is expressed as:

(3) $$\sigma_{c}=\left(1-d_{c}\right)E_{c}\varepsilon_{c}$$

(4) $$d_{c}=\{\begin{array}{c} 1-\frac{\rho_{c}n}{n-1+x^{n}}x\;x\leq 1\\ 1-\frac{\rho_{c}}{\alpha_{c}{\left(x-1\right)}^{2}+x}\;x>1 \end{array}$$

(5) $$\rho_{c}=\frac{f_{c,r}}{E_{c}\varepsilon_{c,r}}$$

(6) $$n=\frac{E_{c}\varepsilon_{c,r}}{E_{c}\varepsilon_{c,r}-f_{c,r}}$$

(7) $$x=\frac{\varepsilon_{c}}{\varepsilon_{c,r}}$$

where $d_{c}$ = damage evolution parameter of the concrete under uniaxial compression, $\;f_{c,r}$ = uniaxial compressive strength of the concrete, $\varepsilon_{c,r}$ = peak compressive strain corresponding to $f_{c,r}\;\mathrm{and}\;\alpha_{c}$ = parameter for descent segment in constitutive relationship of the concrete under uniaxial compression. The value of $\alpha_{c}$ and $\varepsilon_{c,r}$ are listed in Table 1. $\varepsilon_{cu}$ expressed in Table 1 is the ultimate strain of concrete right before the crushing evolution.

The inelastic strains $\tilde{\varepsilon_{c}^{in}}$ of concrete under uniaxial compression corresponding to the compressive stresses $\sigma_{c}$ are used in the CDP model (Figure 2 and Figure 3), which is as follows:

(8) $$\tilde{\varepsilon_{c}^{in}}=\varepsilon_{c}-\varepsilon_{oc}^{el}$$

(9) $$\varepsilon_{oc}^{el}=\frac{\sigma_{c}}{E_{cm}}$$

(10) $$E_{cm}=E_{c}$$

where $E_{cm}$ in the CDP model are equal [41] to $E_{c}$ in the Chinese code for the design of concrete structures [42], which was the modulus of elasticity that was actually adopted in *ABAQUS*.

The plastic strains $\tilde{\varepsilon_{c}^{pl}}$ of concrete under uniaxial compression that must be positive is calculated using an equation as follows:

(11) $$\tilde{\varepsilon_{c}^{pl}}=\tilde{\varepsilon_{c}^{in}}-\frac{D_{c}}{\left(1-D_{C}\right)}\frac{\sigma_{c}}{E_{cm}}\;\tilde{\varepsilon_{c}^{pl}}>0$$

(12) $$D_{c}=1-\sqrt{1-d_{c}\;}\;D_{c}\geq 0$$

where $D_{c}$ (Value of damage parameter of CDP model in ABAQUS based on GB 50010-2010) = damage parameter of concrete under uniaxial compression [43].

### 2.3. Concrete Constitutive Relation under Uniaxial Tension

The constitutive relationship of the concrete under uniaxial tension is expressed as:

(13) $$\sigma_{t}=\left(1-d_{t}\right)E_{c}\varepsilon_{t}$$

(14) $$d_{t}=\{\begin{array}{c} 1-\rho_{t}\left[1.2-0.2x^{5}\right]\;x\leq 1\\ 1-\frac{\rho_{t}}{\alpha_{t}{\left(x-1\right)}^{1.7}+x}\;x>1 \end{array}$$

(15) $$x=\frac{\varepsilon_{t}}{\varepsilon_{t,r}}$$

(16) $$\rho_{t}=\frac{f_{t,r}}{E_{t}\varepsilon_{t,r}}$$

where $d_{t}$ = damage evolution parameter of the concrete under uniaxial tension, $f_{t,r}$ = uniaxial tension strength of the concrete,$\;\varepsilon_{t,r}$ = peak tensile strain corresponding to $f_{t,r}\;\mathrm{and}\;\alpha_{t}$ = parameter for descent segment in constitutive relationship of the concrete under uniaxial tension. The value of $\alpha_{t}$ and $f_{t,r}$ are listed in Table 2.

Same as the previous expression, the cracking strains $\tilde{\varepsilon_{t}^{ck}}$, plastic strains $\tilde{\varepsilon_{t}^{pl}}$ and damage parameter $D_{t}$ of concrete under uniaxial tension, which are used in the CDP model (Figure 2 and Figure 3), are as follows:

(17) $$\tilde{\varepsilon_{t}^{ck}}=\varepsilon_{t}-\varepsilon_{ot}^{el}$$

(18) $$\varepsilon_{oc}^{el}=\frac{\sigma_{t}}{E_{cm}}$$

(19) $$\tilde{\varepsilon_{t}^{pl}}=\tilde{\varepsilon_{t}^{ck}}-\frac{D_{t}}{\left(1-D_{t}\right)}\frac{\sigma_{t}}{E_{cm}}$$

(20) $$D_{t}=1-\sqrt{1-d_{t}\;},\;D_{t}\geq 0$$

where $D_{t}$ (*Value of damage parameter of CDP model in ABAQUS based on GB 50010-2010*) = damage parameter of concrete under uniaxial tension [43].

### 2.4. Crack Detection of CDP in Numeric Analysis

**Figure 4:**
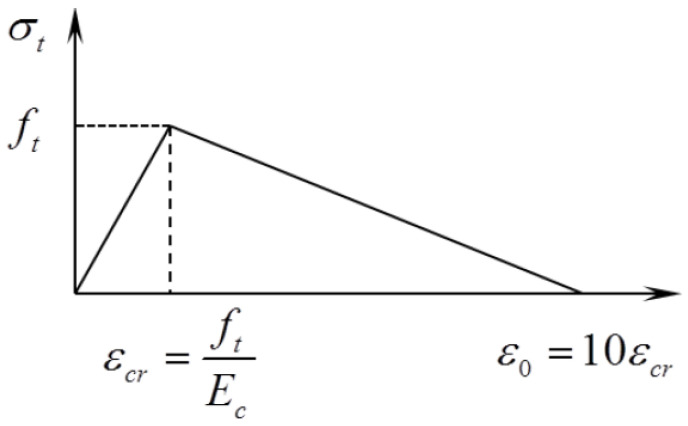
Tension $\sigma_{t}-\varepsilon_{t}$ curve for concrete: linear representation.

The crack, explained by the fact that this crack propagated sideways into a low-stress area in the material and then released energy, would appear after the maximum principal stress reached the concrete tensile strength. Tension stiffening offers concrete the performance that carries tension even after cracking occurs, with tensile strength gradually decreasing. In this study, it presumes that the strain softening linearly reduces the stress to zero at a total strain that is approximately 10 times the failure strain, $\varepsilon_{cr}\;\left(ABAQUS\right)$ (Figure 4).

(21) $$\varepsilon_{cr}=\frac{f_{t}}{E_{c}}$$

The CDP model does not support expressing how the cracks develop. Therefore, in this study, it presumes that cracking occurs at the point when the maximum principal total strain exceeds the value of the strain, $\varepsilon_{o}=10\varepsilon_{cr}$, under which a concrete element totally loses its resistance to tension. For crack detection of joints specimens in this study, $\varepsilon_{o}$ of C50 is 0.001327 and $\varepsilon_{o}$ of C70 is 0.001542.

### 2.5. Material Properties for Reinforcement Bar and Steel Plate

An elastic–plasticity bilinear stress–strain material model was applied to the constitutive relationship of reinforcement bars and steel plates, which could be expressed as:

(22) $$\sigma_{s}=E_{s}\varepsilon_{s},\;\varepsilon_{s}\leq \varepsilon_{y}$$

(23) $$\sigma_{s}=f_{y},\;\varepsilon_{s}\geq \varepsilon_{y}$$

where $E_{s}$ = elastic modulus of reinforcement bar and steel plate, $f_{y}$ = yield strength of the reinforcement bar and steel plate and $\varepsilon_{y}$ = strain at yield strength of reinforcement bar and steel plate.

### 2.6. Numeric Simulation

**Figure 5:**
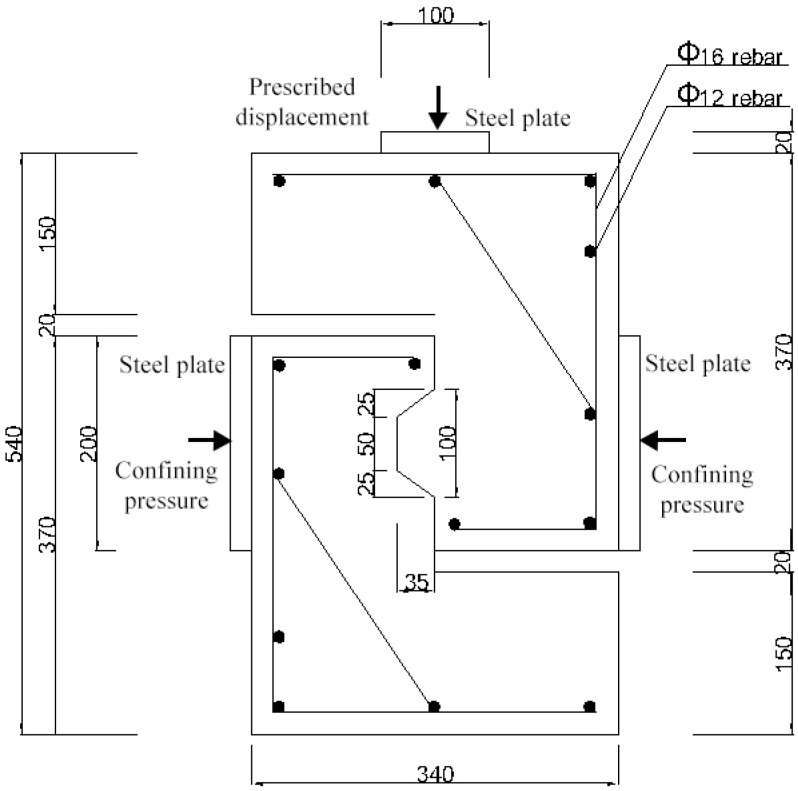
Dimensions of single-keyed dry joints cast in match-casting method tested by Jiang et al. (2016). The unit of measure is millimeter.

In this study, 12 single-keyed dry joints match-casted and tested by Jiang et al. (2016) [31] in the laboratory, two concrete strengths and three confining pressure levels, were analyzed using *ABAQUS*. The overall dimensions of these joints were all 340 × 540 × 100 mm^3^ with 35 mm depth of keys (Figure 5), on which 6 numeric single-keyed dry joints without fixing imperfections of keys were set up based. In the study held by Jiang et al. (2016), 12 specimens of single-keyed dry joints were renamed as Kn−Ha−m, where K indicated keyed dry joint and the number following represents the number of keys, H identified as the type of the concrete (HSC), and the number following was concrete strength (5 represented C50 and 7 represented C70), m represented the confining pressure level. In this study, these specimens were represented as $\left(M/L/R\right)+\left(\;F_{i,\;i=0,\;1,2}\;\right)Kn-Ha-m$ to get unified, where (M/L/R) represented the way how the lower surfaces of male key contacted with the female part before loading [M: in parallel; L: at the left bottom of female key; R: at the right bottom of female key (Figure 1)], $F_{i}$ represents that the location of fixing imperfections of male key [$F_{0}$ (as both L and R only accompany with $F_{0}$ in this study, both $LF_{0}$ and $RF_{0}$ are simply named as L and R): at lower surface; $F_{1}$: at upper surface (area of the root);$\;F_{2}$: at vertical surface]. In the below illustration, these fixing imperfections are presented as L, R, $MF_{1}\;\mathrm{and}\;MF_{2}$ respectively. For example, specimen *MK1-H5-0.5* is without fixing imperfections and its concrete strength is 50 MPa under confining pressure of 0.5 MPa; specimen *RK1-H5-0.5* has fixing imperfections at lower surface of male key that contacts with female key at right bottom; specimen *MF_1_K1-H5-0.5* has fixing imperfections at upper surface of male key.

**Figure 6:**
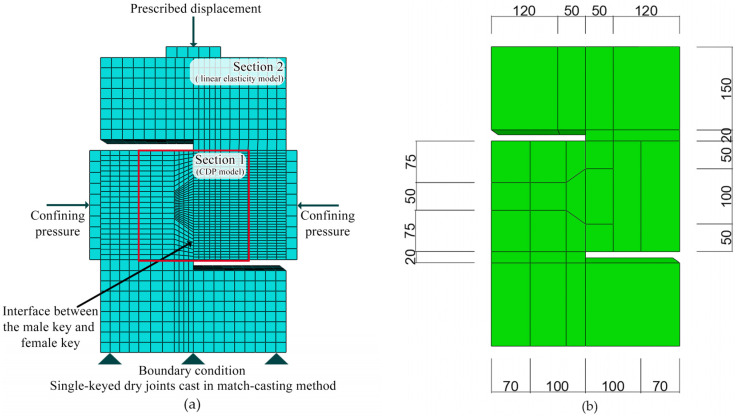
Finite-element model (concrete-damaged plasticity and linear elasticity model), including (**a**) mesh and boundary conditions; (**b**) partition cells. The unit of measure is millimeter.

To simplify, the concrete parts of each specimen were divided into two kinds of sections, the CDP model (Section 1, 10 cells considered in the mesh) and the linear elasticity model (Section 2, 17 cells considered in the mesh), respectively *(*Figure 6). In order to better simulate, a solid model was put into the study (Figure 6), in which the concrete and the reinforcement bars were modeled as solid elements and truss elements, respectively. The approximate element size of male key was 5 mm, and that of female key and the elements adjacent to the male key was 10 mm, while that of the rest was 20 mm. The total number of elements was 11,546 (11,220 linear hexahedral elements of type C3D8R and 326 linear line elements of type T3D2) and the total number of nodes are 14,128. In the Interaction model, all the concrete parts were designated as the host region, and all reinforcement bars were modeled as the embedded region in concrete by using Embedded region constraint. This approach provides a means of setting rebar elements at designed locations with the constraints on a translational degree of freedom equal to that of the host elements surrounding them. The steel bars were arranged as shown in Figure 5. The thickness of the reinforcement cover was 20 mm.

Surface-to-surface contact discretization provided in *ABAQUS* was taken for formulating the contact simulation for interfaces between two segments as well as interfaces between steel plates and specimens (Figure 5), and finite sliding analysis procedure that allows for arbitrary relative slippage and rotation between contact surfaces was used in this analysis (*ABAQUS*) as the relative sliding or rotational momentum between two contact surfaces is large. Surfaces of the female parts with larger surfaces area were defined as master surface while surfaces of the male parts were defined as the slave surface. Surfaces of steel plates with higher stiffness were taken as master surface while surfaces of the specimens were taken as slave surface. The friction coefficient for the contact surfaces was set as 0.6 based on the experimental results and recommendations [24,39,44,45]. Particularly, material nonlinear and contact nonlinear analyses were used in the numeric simulations.

Two general-static steps were set up for the process of loading. By applying constant uniform pressure on left and right side steel plates, the confining pressure covering an area of 100 × 200 mm^2^ (Figure 5) was simulated and assigned to Step 1, including 0.5-, 1.0- and 2.0-MPa levels to investigate the shear behavior of single-keyed dry joints under lower prestress that was disadvantageous and the effect of lower confining pressure on that [7,10,11,12,20,28,29,30,31,46]. A displacement-controlled load was assigned to Step 2 and applied on a reference point to which the coupling of the top surface of the upper steel plate was bonded (Figure 5). This displacement-controlled load was simulated by creating a boundary condition moving vertically downward and set as a value of 4 mm. The bottom surface of each specimen was restrained against all translational degrees of freedom (Figure 6a), while the reference point illustrated previously was restrained against the displacement in x- and z-direction, which was assigned to the Initial step. This setup made the shear plane of joints to bear pure shear load without flexural moment.

## 3. FE Analysis Results

### 3.1. Shear Capacity

Table 3 compares the shear strength of single-keyed dry-joint specimens in terms of experimental tests from Jiang et al. (2016) [31] and numeric analysis in this study, with each group of cases concerning two experimental specimens and one numeric specimen. The numeric data agreed well with the corresponding experimental results as the average and standard deviation are approximately 98.0% and 7.8%, respectively. Overall, the FE model proposed in this study was reliable to predict the ultimate shear strength of single-keyed dry joints.

### 3.2. Load–Displacement Relationship

**Figure 7:**
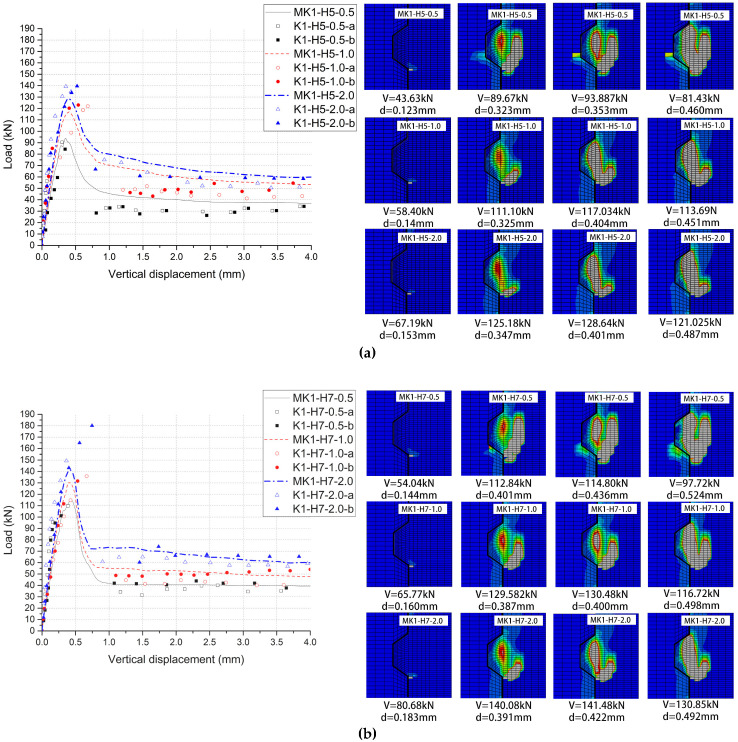
Load–displacement curve and crack patterns from numeric analysis. (**a**) Specimens *MK1-H5-0.5*, *MK1-H5-1.0* and *MK1-H5-2.0*; (**b**) specimens *MK1-H7-0.5*, *MK1-H7-1.0* and *MK1-H7-2.0*.

**Figure 8:**
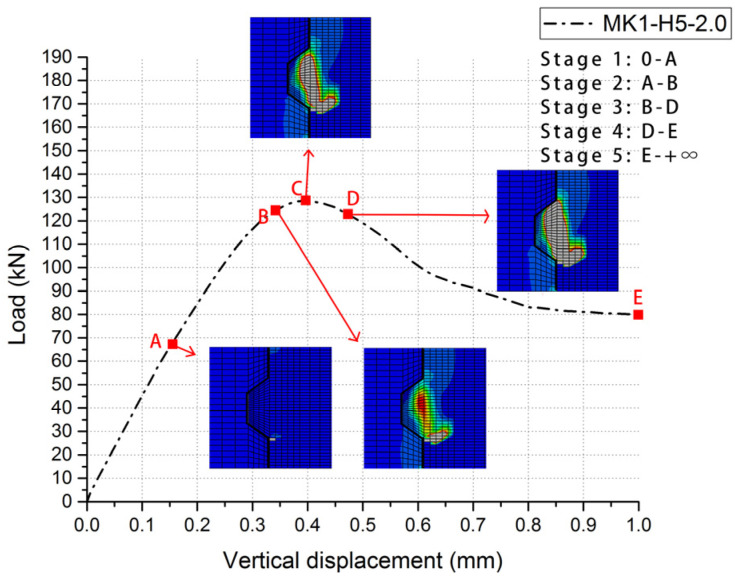
Five stages in load–displacement curve of numeric single-keyed dry joints.

The comparisons of the load–displacement relationships of 6 groups of cases were shown in Figure 7, which indicates the main features of them and the significant similarity between experimental results and numeric data of each group. The experimental results from Jiang et al. (2016) are presented as scattered dots and numeric data from the FE model in *ABAQUS* are shown as lines. Due to a lack of consistent collection of data in experimental tests, it could be easy to ignore some information from those load–displacement dots. One of that was to distinguish each stage in the load–displacement relationships. However, numeric analysis provides a better tool to figure out this problem. Overall, all these numeric load–displacement curves could be divided into five stages (Figure 8).

In Stage One, the linear elastic state, no crack happened in the key area of the joint specimen and the relative displacement increased linearly when the applied load rose. In Stage Two, the nonlinear elastic-plastic state, as the first crack occurred and the specific value and pattern were shown in Table 3 and Figure 7 respectively, the stiffness of joints (the slope of the curve) decreased, which led to nonlinearly increased relative displacement with the climbing load. In Stage Three, the failure state, on the moment the shear-off cracks appeared in the male key area of the joint specimen, the slope of curve slumped to zero and the load reached the maximum value at the same time. As overwhelming plastic deformation and substantial material damage were evident, the shear-off cracks ran through the entire male keys of the joint specimen and the sustained load of specimen suddenly dived after the maximum load occurred. In Stage Four, the descending phase, the load went into free-fall as the shear-off cracks had already run through at the root of key and sheared off the entire key from the male part. This large slip could be illustrated by the processes of cracking, leading to the maximum resistance of concrete [10,31]. In Stage Five (the residual phase), the shear capacity of the joint specimen finally decreased to a constant value defined as residual load, mainly provided by friction action of the shear plane relative to the horizontal confining pressure level and friction coefficient. Stage one, two and five roughly followed the same trend in both tests [31] and simulations. Differing from the experimental results, it could be more obvious to distinguish Stage three (the failure state) and Stage four (descending phase) in *ABAQUS*. That processes of cracks in Stage Four were too sudden to record with vertical displacements and loads [31].

### 3.3. Crack Patterns

**Figure 9:**
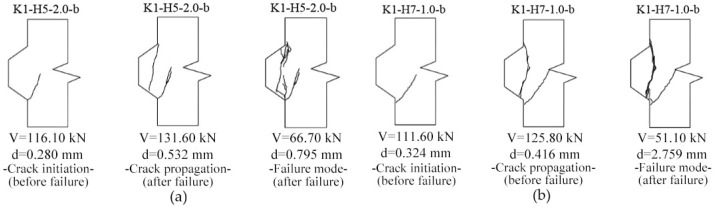
Crack evolution history of single-keyed dry joints specimen tested by Jiang et al. (2016) [31]. (**a**) Specimen *K1-H5-2.0-b*; (**b**) specimen *K1-H7-1.0-b*.

Figure 7 provides the crack patterns associated with tension strains of $\varepsilon_{o}$ (Figure 4) for these 6 numeric specimens of joints. Gray areas in the pictures represent the cracks propagated and nonlinear concrete material. By numeric analysis in single-keyed dry joints, Table 3 shows the angles at which the cracks propagated and the loads when the cracks occurred. These angles were measured from the angle from the element that exceeded the value of $\varepsilon_{cr}$ at the left bottom to that at the top right corner. From experimental results [31] (Figure 9), the angles of the first crack and the vertical crack were approximately 55 degrees and 90 degrees, respectively, and the crack patterns before failure as well as after failure were basically as same as those from numeric results (Figure 7). Overall, comparing crack evolutions obtained from this numeric analysis to those from experiments, they are highly similar, further indicating that the FE model of single-keyed dry-joint specimens is reliable in this study.

These numeric crack evolution histories express how the load–displacement relationships of specimens changed five continuous stages in this study. For specimens *MK1-Ha-m (a = 5 and 7; m = 0.5, 1.0 and 2.0)* without fixing imperfections, when the load reached to approximately 50.5% of the shear capacity, the first crack initiated at the right bottom of the male key, which presented the beginning of nonlinear elastic–plastic state (Stage two) of load–displacement relationships of the male key. Then the cracks initiated at the right bottom of male key propagated upward at approximately 45 degrees respective to the horizontal, and this crack ceased to grow before the failure state (Stage three). Later on, when the load rose to averagely 97.4% of the shear capacity, the shear-off cracks leading to Stage Three initiated from the middle of the root of the male key and propagated vertically upwards and downwards through the sheared plane. The applied load reached to the shear capacity of the specimen before shear-off cracks totally ran through the male key. In the descending phase (Stage four), the male key was sheared off. The interval from Stage three to Stage four was very short, therefore, experimental cracking patterns lacked those figures of that interval as it was hard to record [31] but numeric study can easily output those crack patterns throughout the whole failure process. From Stage four to Stage five (the residual phase), as the concrete has already lost the resistance, two parts of those specimens kept as a whole by the confining pressure, and the residual load was mainly provided by friction action of shear plane.

## 4. Parametric Study: Fixing Imperfections in Shear Key

A parametric study of fixing imperfections of key was proposed based on the validated FE model. For each group of cases with the same confining pressure level and same concrete strength, there are one case without fixing imperfections and four different fixing imperfections (Table 4) corresponding to Figure 1, which was presented as “without fixing imperfections”, “with fixing imperfections at upper surface of male key only $\left(MF_{1}\right)$”, “with fixing imperfections at vertical surface of male key only ($MF_{2}$)”, “with fixing imperfections at lower surface of male key that contacts at right bottom before loading (R)”, and “with fixing imperfections at lower surface of male key that contacts at left bottom before loading (L)”, in total. All of the dimensions of fixing imperfections were set as 0.5 mm.

### 4.1. Effects of Fixing Imperfections of Keys on Shear Capacity and Load–Displacement Relationship

**Figure 10:**
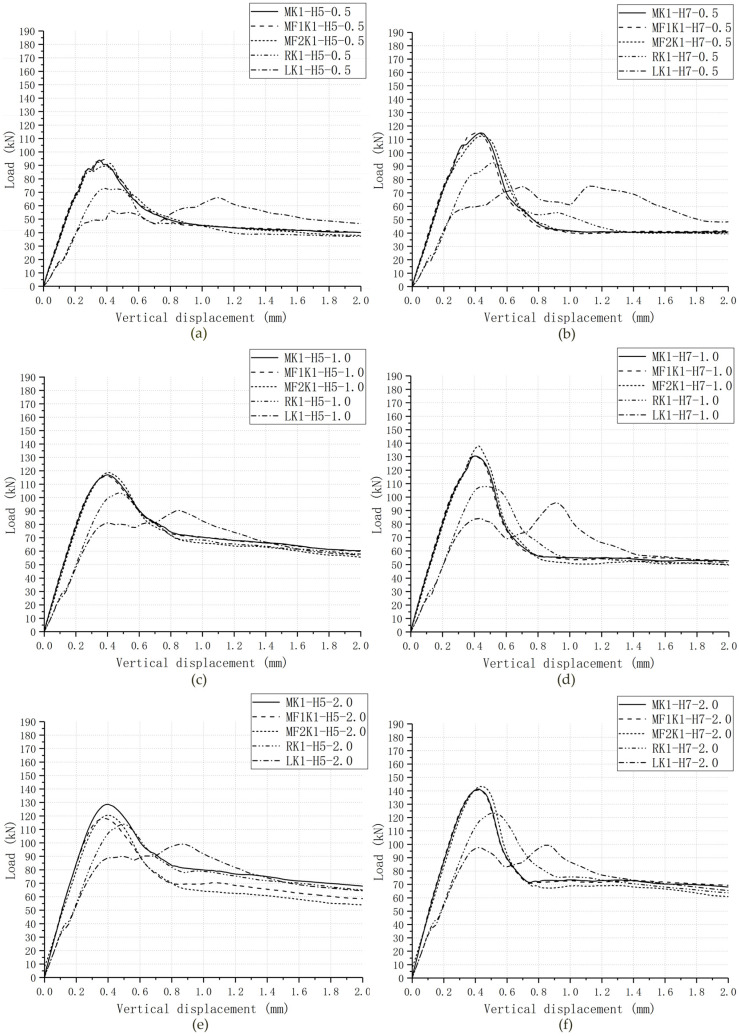
Numeric load–displacement curves for C70 and C50 specimens under various confining pressure. (**a**) C50 specimens under 0.5 MPa; (**b**) C70 specimens under 0.5 MPa; (**c**) C50 specimens under 1.0 MPa; (**d**) C70 specimens under 1.0 MPa; (**e**) C50 specimens under 2.0 MPa; (**f**) C70 specimens under 2.0 MPa.

Table 5 and Figure 10 present that the four fixing imperfections of key affected shear capacity and load–displacement relationship of single-keyed dry-joint specimens, respectively. Overall, L and R had a great impact on shear capacity and load–displacement relationship, while $MF_{1}$ and $MF_{2}$ did not significantly affect them.

The changes in shear capacity compared with joints specimens without fixing imperfections were illustrated in Table 5. In terms of single-keyed dry-joint specimens with L and R, especially with L, shear capacity decreased significantly. On the other hand, the effects of $MF_{1}$ and $MF_{2}$ on shear capacity were both slight. As for C50 specimens under 0.5 MPa confining pressure level, the shear capacity of specimens *MF_1_K1-H5-0.5*, *MF_2_K1-H5-0.5*, *RK1-H5-0.5* and *LK1-H5-0.5* was reduced by −0.682%, 2.38%, 22.2% and 29.7%, respectively, compared with specimen *MK1-H5-0.5*. Similar behavior was observed for other specimens.

The changes in load–displacement relationships compared with joints specimens without fixing imperfections were illustrated in Figure 10. In terms of single-keyed dry-joint specimens with $MF_{1}$, the overall trend of load–displacement relationship did not change, but initial stiffness reduced slightly due to lower monolithic stiffness of male key, which was the same as the joints specimens with $MF_{2}$. In terms of single-keyed dry-joint specimens with L and R, initial stiffness was much lower, which was one of the reasons why shear capacity considerably decreased, and vertical displacement relative to ultimate shear strength considerably increased. In particular, as for those joints with L the load would experience a drop but increase slightly soon after the shearing-off cracks occurred. These fluctuations could repeat at least once, and the ultimate shear strength arose at the final peak load. That could be explained for one of the reasons why single-keyed dry-joint specimens with L experienced the lowest ultimate shear strength and the most different load–displacement relationships than those single-keyed dry joints with other fixing imperfections.

### 4.2. Effects of Fixing Imperfections of Keys on Crack Patterns

**Figure 11:**
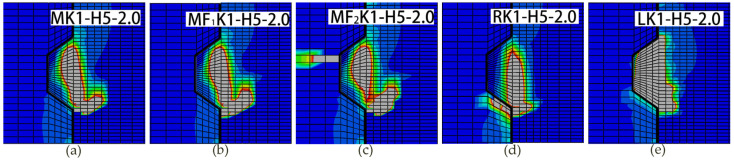
Crack patterns from numeric analysis for joints specimens K1-H5-2.0 under different fixing imperfections of keys when they reached the ultimate shear strength. (**a**) Vu= 128.64 kN; (**b**) Vu= 118.29 kN; (**c**) Vu= 120.60 kN; (**d**) Vu= 113.95 kN; (**e**) Vu= 99.14 kN.

Figure 11 compares the crack patterns at the applied load that reached to the ultimate shear strength as for specimens *(M/MF_1_/MF_2_/R/L)K1-H5-2.0.* Overall, the crack patterns of single-keyed dry joints with fixing imperfections differed from the shearing-off failure mode of specimen *MK1-H5-2.0* to different degrees. Similar behavior was observed for other specimens.

The overall crack patterns and shearing-off failure mode of specimen *MF_1_K1-H5-2.0* were similar to those of specimen *MK1-H5-2.0*, but with much propagated diagonal cracks at the right bottom of the male key. As for specimen *MF_2_K1-H5-2.0*, the initial crack occurred at the left top corner of the female key before the diagonal cracks initiated at the right bottom of the male key, while the crack patterns and failure mode in male part were similar to those of specimen *MK1-H5-2.0*. Similarly, the initial crack of specimen *RK1-H5-2.0* occurred at the right bottom corner of the female key before the diagonal cracks initiated at the right bottom of the male key. However, the diagonal cracks propagated much upward and the shearing-off cracks initiating at the root of male key propagated at more like 90 degrees respective to the horizontal. As for specimen *LK1-H5-2.0*, the first crack initiated at the middle of the lower surface of male key and then propagated upward at approximately 45 degrees respective to the horizontal, then the second crack initiated at the middle area in the male key and propagated at approximately 60 degrees respective to the horizontal until sheared off the male key from left bottom to right top. The first peak load-in-load–displacement curves occurred right after these shearing-off curves appeared. As the shearing-off cracks evolved in almost the whole key area until the key was sheared-off, the load would rise slightly again at least once. Through this process of cracking, more concrete elements attended to sustain the shear load. Therefore, the ultimate shear strength arose at the last.

These simulation crack patterns were also similar to the experimental results from [15,23,30,31,39], especially the crack patterns of specimen *MF_2_K1-H5-2.0* [30], specimen *LK1-H5-2.0* [31,39] and specimen *RK1-H5-2.0* [15,30], though many other experimental studies pointed out that no obvious cracks propagated in the female part of keyed joints [1,7,10,11,12,20,28,29,30] as specimen *MK1-H6-2.0* and specimen *MF_1_K1-H5-2.0* in Figure 11a,b. Most of the cracks in shear key [15,23,30,31,39] resemble the simulating crack patterns of specimen *LK1-H5-2.0* and specimen *RK1-H5-2.0*.

### 4.3. Effects of Fixing Imperfections of Keys on the Contour Plot of the Maximum Principal Stress Contour

**Figure 12:**
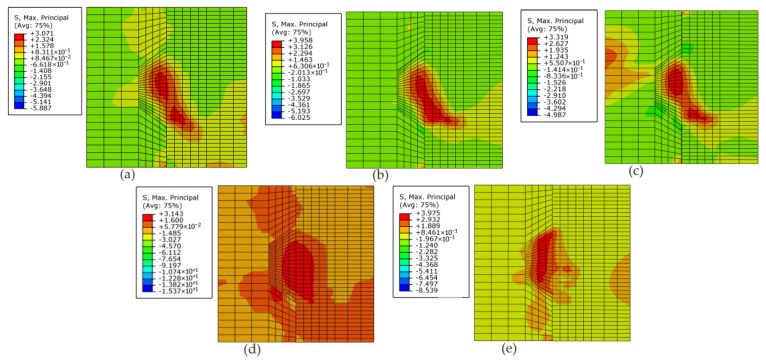
Contour plot of the maximum principal stress contour of specimens K1-H5-2.0 with different fixing imperfections of keys. (**a**) specimen *MK1-H5-2.0*; (**b**) specimen *MF_1_K1-H5-2.0*; (**c**) specimen *MF_2_K1-H5-2.0*; (**d**) specimen *RK1-H5-2.0*; (**e**) specimen *LK1-H5-2.0*.

Figure 12 compares the contour plot of the maximum principal stress at the applied load of 90 kN as for specimens (*M/MF_1_/MF_2_/R/L*)*K1-H5-2.0*. Overall, compared with the specimen *MK1-H5-2.0* without fixing imperfections of key, the level of maximum principal stress arising at the male key of specimens *MF_1_/MF_2_/R/L*)*K1-H5-2.0* rose, and the stress concentrations corresponding to the crack patterns changed. That could be explained by the condition of fixing imperfections of key, leading to different shear transfer mechanisms from the male part to the female part. Those different shear transfer mechanisms reduced load transferred from the lower surface of the male key to the female part and facilitated stress concentrations, hence decreased shear capacity. Similar behavior was observed for other specimens.

The maximum principal stress in stress concentration areas of specimens (*M/MF_1_/MF_2_/R/L*)*K1-H5-2.0* were 3.071, 3.958, 3.319, 3.143 and 3.975 MPa, respectively, which indicated that higher maximum principal stress reduced load transferred from male part to female part through the lower surface of the male key at the same load of 90 kN. The stress concentration of specimen *MK1-H5-2.0* was found in the root and the right bottom of the male key. The overall contour plot of maximum principal stress contour of male part of specimen *MF_1_K1-H5-2.0* as well as specimen *MF_2_K1-H5-2.0* was similar to specimen *MK1-H5-2.0*. However, under confining pressure and vertical load at the same time, the top left corner of the female key of specimen *MF_2_K1-H5-2.0* sustained initial tensile stress concentration, losing much capacity of tension resistance of concrete elements as well. The contour plot of maximum principal stress contour of specimen *RK1-H5-2.0* and that of specimen *LK1-H5-2.0* totally differed from that of specimen *MK1-H5-2.0*. On one hand, the stress concentration areas (3.143 MPa) of specimen *RK1-H5-2.0* exhibited at the bottom of the female key before those exhibited at the root area of the male key. The stress concentration areas where the diagonal cracks propagated were unobvious. On the other hand, the stress concentration of specimen *LK1-H5-2.0* (3.975 MPa) was markedly found in nearly the whole male key area. Among these shear transfer mechanisms, it can be seen that those occurring in single-keyed dry joints with fixing imperfections at lower surface (L and R), especially L, were the most unfavorable condition for shear capacity. Hence, the assumption proposed by Zhou et al. (2005) [10] that the shear capacity of each shear key in multiple-keyed dry joints with a higher possibility of fixing imperfections could not fully develop due to the nonuniformly distributed shear load was not absolutely substantial. However, each shear key in single-keyed dry joints sustained the same load in this parametric study of fixing imperfections, they did not have the same shear transfer mechanisms from the male part to the female part and ultimate shear strength.

## 5. Parametric Study: Confining Pressure

**Figure 13:**
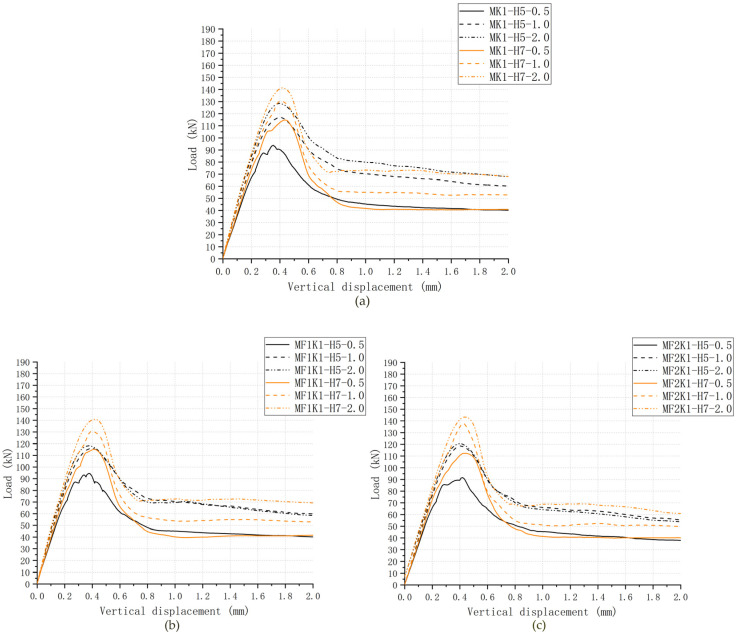

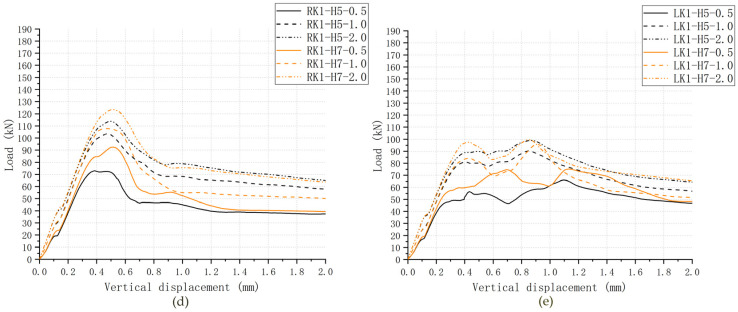
Load–displacement curves for single-keyed dry joints with fixing imperfections of keys under confining pressure of 0.5–2.0 MPa. (**a**) Specimens *MK1-Ha-m*; (**b**) specimens *MF_1_K1-Ha-m*; (**c**) specimens *MF_2_K1-Ha-m*; (**d**) specimens *RK1-Ha-m*; (**e**) specimens *LK1-Ha-m*.

**Figure 14:**
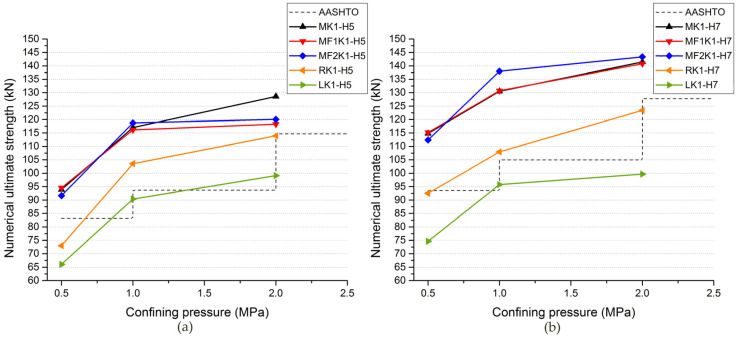
Effect of fixing imperfections of keys on shear capacity and comparisons between numeric values of shear capacity and AASHTO formula of C50 (**a**) and C70 (**b**) specimens under various confining pressure levels. (**a**) C50 specimens; (**b**) C70 specimens.

**Figure 15:**
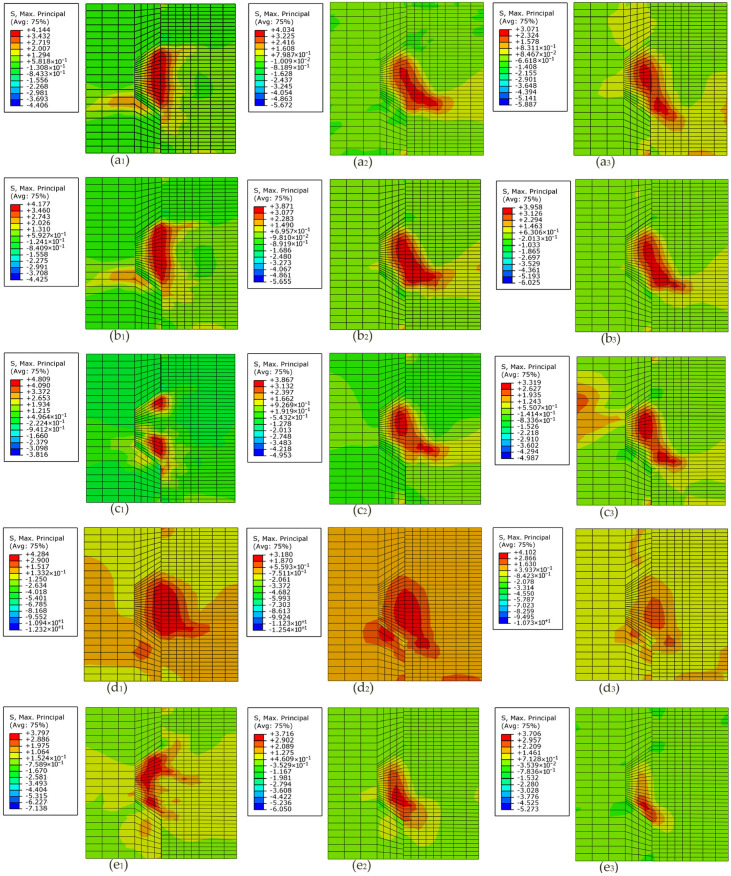
Contour plot of the maximum principal stress contour of specimens K1-H5-m with different fixing imperfections of keys under confining pressure varied from 0.5 MPa to 2.0 MPa. (**a_1_**) Specimen *MK1-H5-0.5* (**a_2_**) specimen *MK1-H5-1.0* (**a_3_**) specimen *MK1-H5-2.0*; (**b_1_**) specimen *MF_1_K1-H5-0.5*; (**b_2_**) specimen *MF_1_K1-H5-1.0*; (**b_3_**) specimen *MF_1_K1-H5-2.0*; (**c_1_**) specimen *MF_2_K1-H5-0.5*; (**c_2_**) specimen *MF_2_K1-H5-1.0*; (**c_3_**) specimen *MF_2_K1-H5-2.0*; (**d_1_**) specimen *RK1-H5-0.5*; (**d_2_**) specimen *RK1-H5-1.0*; (**d_3_**) specimen *RK1-H5-2.0*; (**e_1_**) specimen *LK1-H5-0.5*; (**e_2_**) specimen *LK1-H5-1.0*; (**e_3_**) specimen *LK1-H5-2.0*.

From Table 3 and Figure 7, as for single-keyed dry-joint specimens without fixing imperfections of key (specimens *MK1-H5-m* and *MK1-H7-m*), it can be seen that the higher confining pressure level (0.5 MPa, 1.0 MPa and 2.0 MPa) was, the later linear elastic state end (when the first crack arose), the higher initial stiffness was, and the lower angle at which the area of the cracks in male part propagated to the horizontal and the higher shear capacity was, with restrained cracks areas after failure, corresponding to the experimental conclusion from Liu et al. (2018) [23]. Table 5, Figure 13, Figure 14 and Figure 15, present the effect of confining pressure varied from 0.5 MPa to 2.0 MPa on shear capacity, load–displacement relationship and crack patterns of single-keyed dry joints with fixing imperfections of key compared with specimens *MK1-Ha-m*.

### 5.1. Effects of Confining Pressure on Shear Capacity and Load–Displacement Relationship

For single-keyed dry-joint specimens with fixing imperfections of key, both ultimate shear strength and initial stiffness (Figure 13) raised with confining pressure increasing. These trends of shear strength were more evident when confining pressure increasing from 0.5 MPa to 1.0 MPa, which was the same as specimens without fixing imperfections of key (Figure 14).

Table 5 compares the negative effect of fixing imperfections of key on ultimate shear strength and finds out that the shear capacity of those specimens decreased much caused by fixing imperfections of key when confining pressure was 0.5 MPa. This is to say, higher confining pressure (1.0 MPa and 2.0 MPa) was good for alleviating the negative effect of fixing imperfections of key on shear capacity and it was more evident when confining pressure increasing from 0.5 MPa to 1.0 MPa. For example, the ultimate shear strength of specimen *LK1-H5-0.5* reduced by 29.7%, that of specimen *LK1-H5-1.0* reduced by 22.8%, and that of specimen *LK1-H5-2.0* reduced by 23.0%. Similar behavior was observed for other specimens.

### 5.2. Effects of Confining Pressure on the Contour Plot of the Maximum Principal Stress Contour

Figure 15 (a_i_, b_i_, c_i_, d_i_ and e_i_, i = 1,2,3) shows the contour plot of the maximum principal stress contour of specimens *MK1-H5-m_i_, MF_1_K1-H5-m_i_, MF_2_K1-H5-m_i_, RK1-H5-m_i_* and *LK1-H5-m_i_* (*m_i_* = 0.5, 1.0 and 2.0) at the applied load of 90 kN (a_i_), 90 kN (b_i_), 90 kN(c_i_), 70 kN (d_i_) and 50 kNc(e_i_), respectively.

Basically, as confining pressure increased, the maximum principal stress-in-stress concentration areas decreased, indicating that much load was transferred from the male part to the female part by the lower surface of the key. The regions of stress concentration were in accord with the crack patterns. Therefore, cracking was restrained, and shear capacity was higher. In addition, the regions where stress concentration occurred changed, which indicated different shear transfer mechanism with lower angles at which the cracks propagated to the horizontal and changed quantity of concrete elements where stress concentration occurred. As confining pressure increased, for specimens *RK1-H5-m_i_* and *LK1-H5-m_i_*, the number of concrete elements where stress concentration occurred were fewer, whereas that was almost unchanged in the cases of specimens *MK1-H5-m* and *MF_1_K1-H5-m*. The opposite was the case of specimen *MF_2_K1-H5-m*, as those concrete elements of stress concentration of specimens *MF_2_K1-H5-0.5* were the fewest. In addition, higher confining pressure led up to a more obvious stress concentration at the top left corner of the female key in the cases of specimens *MF_2_K1-H5-m*. Similar behavior was observed from other specimens.

## 6. Parametric Study: Concrete Strength

**Figure 16:**
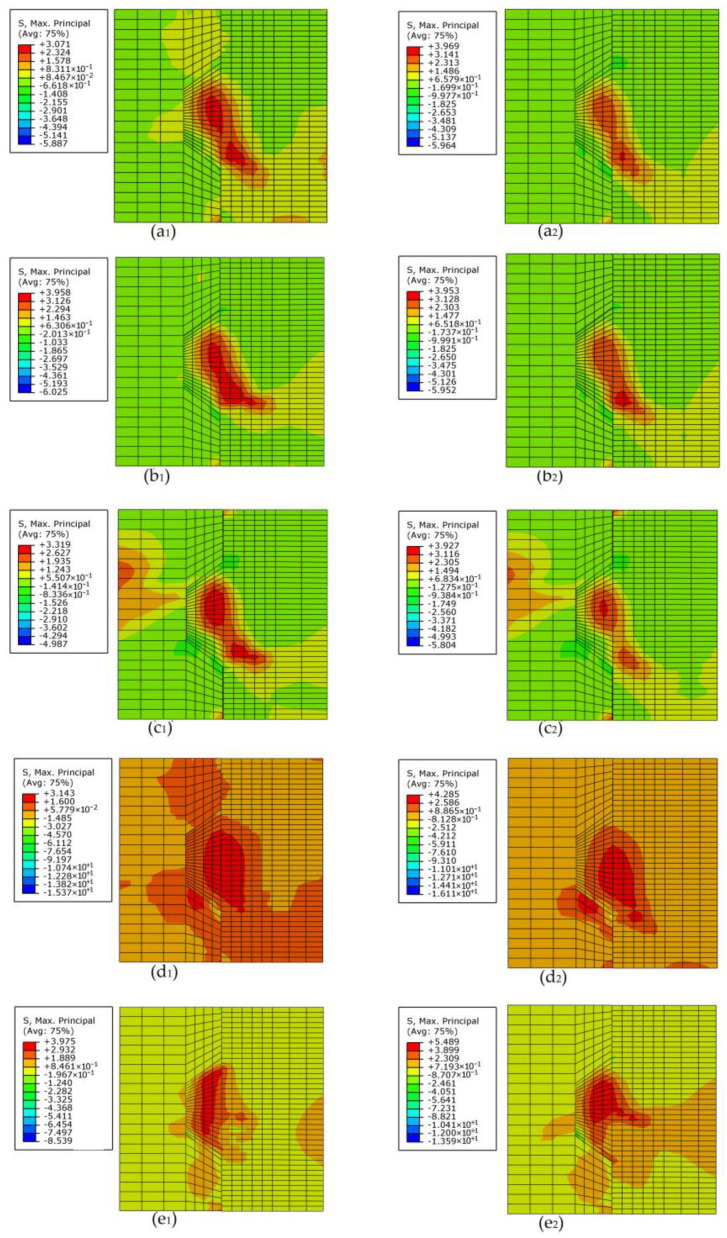
Contour plot of the maximum principal stress contour of specimens *K1-H5-2.0* (left) and *K1-H7-2.0* (right) with different fixing imperfections of keys. (**a_1_**) Specimen *MK1-H5-2.0* (**a_2_**) specimen *MK1-H7-2.0*; (**b_1_**) specimen *MF_1_K1-H5-2.0*; (**b_2_**) specimen *MF_1_K1-H7-2.0*; (**c_1_**) specimen *MF_2_K1-H5-2.0*; (**c_2_**) specimen *MF_2_K1-H7-2.0*; (**d_1_**) specimen *RK1-H5-2.0*; (**d_2_**) specimen *RK1-H7-2.0*; (**e_1_**) specimen *LK1-H5-2.0*; (**e_2_**) specimen *LK1-H7-2.0*.

From Table 3 and Figure 7—as for single-keyed dry-joint specimens without fixing imperfections of key (specimens *MK1-Ha-0.5*, *MK1-Ha-1.0*, *MK1-Ha-2.0*)—it can be seen that the higher concrete strength (C50 to C70) was, the later linear elastic state terminated, the higher initial stiffness was, and the lower angle at which the cracks area in male part propagated to the horizontal and the higher shear capacity was, with restrained the area of the cracks after failure. Table 5, Figure 13 and Figure 16 present the effect of concrete strength varied between C50 and C70 on shear capacity, load–displacement relationship and crack patterns of single-keyed dry joints with fixing imperfections of key compared with specimens *MK1-Ha-m*.

### 6.1. Effects of Concrete Strength on Shear Capacity and Load–Displacement Relationship

For single-keyed dry-joint specimens with fixing imperfections of key, both ultimate shear strength and initial stiffness (Figure 13) raised with concrete strength increasing. Table 5 compares the negative effect of fixing imperfections of key on ultimate shear strength and finds out that the shear capacity of that specimens reduced much caused by $MF_{1}$ and $MF_{2}$ when concrete strength was C50 and the opposite case of specimens with L and R. For example, the ultimate shear strength of specimen *MF_1_K1-H5-2.0* reduced by 8.05%, while that of specimen *MF_1_K1-H7-2.0* reduced by 0.474%; ultimate shear strength of specimen *LK1-H5-2.0* reduced by 23.0%, while that of specimen *LK1-H7-2.0* reduced by 29.5%. Similar behavior was observed for other specimens.

### 6.2. Effects of Concrete Strength on the Contour Plot of the Maximum Principal Stress Contour

Figure 16 (a_i_, b_i_, c_i_, d_i_ and e_i_, i = 1,2) shows the contour plot of the maximum principal stress contour of specimens *MK1-Ha_i_-2.0*, *MF_1_K1-Ha_i_-2.0*, *MF_2_K1-Ha_i_-2.0*, *RK1-Ha_i_-2.0* and *LK1-Ha_i_-2.0* (*a*_i_ = 5 and 7) at the applied load of 90 kN, respectively. As concrete strength was higher, the regions where the stress concentration occurred changed slightly and the number of concrete elements where the stress concentration occurred were fewer, which were relative to the lower angles at which the cracks propagated to the horizontal and the restrained cracks, respectively. The regions of stress concentration were accord with the numeric crack patterns. In addition, though the maximum principal stress in stress concentration areas increased (except that specimens *MF_1_K1-H5-2.0* and *MF_1_K1-H7-2.0* were almost the same) as concrete strength increased, the concrete tensile strength was improved as well, which was relative to that the load transferred from the lower surface of the male key to female part was less, but the shear capacity was higher. The maximum principal stress in stress concentration areas of specimens (*M/MF_1_/MF_2_/R/L*)*K1-H5-2.0* were 3.071, 3.958, 3.319, 3.143 and 3.975 MPa, respectively, while those of specimens *(M/MF_1_/MF_2_/R/L)K1-H7-2.0* were 3.969, 3.953, 3.927, 4.285 and 5.489 MPa, respectively, which could be generalized that the load transferred from male part to female part through the lower surface of the male key of specimen *MF_1_K1-H5-2.0* and specimen *MF_2_K1-H5-2.0* decreased more dramatic than specimen *MF_1_K1-H7-2.0* and specimen *MF_2_K1-H7-2.0* and the opposite case of specimens with L and R.

## 7. Comparisons between Numeric Analysis and AASHTO

According to the AASHTO [24], the shear capacity of keyed dry joints includes two parts, the friction force relative to confining stress levels between the flat surfaces, and the shear contribution of keys relative to both the concrete strength and the confining stress levels. The formula proposed by AASHTO [24] is as follow:

(24) $$V_{a}=A_{K}\sqrt{6.792\times 10^{-3}f_{cm}}\left(12+2.466\sigma_{n}\right)+0.6A_{sm}\sigma_{n}$$

where: $A_{K}\;$= area of the root of all keys in the failure plane ($mm^{2}$); $f_{cm}\;$= characteristic compressive strength of concrete (MPa); $\sigma_{n}$=average compressive stress in concrete across the key root area (MPa); $A_{sm}\;$= area of contact between flat surfaces on the failure plane ($mm^{2}$). The value of 0.6, suggested by AASHTO [24], represents the friction coefficient between concrete-to concrete surfaces.

Table 5 and Figure 14 compare single-keyed dry joints, C50 and C70, with or without fixing imperfections in terms of numeric analysis and AASHTO formula under confining pressure levels of 0.5 MPa, 1.0 MPa and 2.0 MPa, respectively and Table 5 calculated the deviations (error) between them. For specimens *MK1-Ha-m*, specimens with $MF_{1}$ and specimens with $MF_{2}$, the values of shear capacity in this numeric analysis were much higher than those from AASHTO formula at least 5.20%. For specimens with fixing imperfections with L and R, values of shear capacity in this numeric analysis were much lower than those from AASHTO formula ranging from 1.05% to 22.0%, except that the numeric values of specimen *RK1-H5-1.0* and specimen *RK1-H7-1.0* were higher than the values from AASHTO 10.5% and 2.84%, respectively. The errors (Table 5) of single-keyed dry joints with L (specimens *LK1-Ha-m*) between numeric shear capacity and calculation from AASHTO, was the most significant deviations that overestimate the shear capacity of single-keyed dry joints. Basically, the error in the ultimate strength predicted from the numeric model and AASHTO formula was the lowest for the confining pressure of 2.0 MPa. Particularly, as for specimens with L and R, the predicted ultimate strength from AASHTO formula was not always conservative and even worse for the confining pressure of 2.0 MPa. For example, as for specimen *MK1-H5-0.5* and specimens *MK1-H5-2.0*, those errors decreased from 12.8% to 12.2%, while for specimen *LK1-H7-0.5* and for specimens *LK1-H7-2.0*, those adverse errors increased from 20.2% to 21.1%. On the contrary, those errors for the confining pressure of 1.0 MPa were mostly the highest. Generally, the AASHTO formula tended to underestimate the shear capacity of single-keyed dry joints for the confining pressure of 0.5 MPa and 1.0 MPa much often except for specimens with L and R, while it was not enough conservative for the confining pressure of 2.0 MPa or even higher that needs to further study, especially for single-keyed dry joints with fixing imperfections.

### 7.1. A Correction Factor of Direct-Shear Strength Based on AASHTO and This Numeric Study

Zhou et al. (2005) suggested that a correction factor of direct-shear strength should be provided in the formula in AASHTO for predicting the shear capacity of multiple-keyed dry joints with a higher possibility of fixing imperfections [10]. In this study, it can be seen that the shear behavior of single-keyed dry joints with fixing imperfections changed and the shear capacity decreased in different degrees. Whereas, the specific fixing imperfections of keys for multiple-keyed dry joints are hard to confirm. Assuming that the shear friction force is basically unchanged, the reliability of the correction factor defined as 0.90 (Table 6) for the direct-shear strength was deduced on the basis of an average ratio [Value two (the average numeric direct-shear strength for single-keyed dry-joint specimens with and without fixing imperfections)/ Value one (the numeric direct-shear strength for single-keyed dry-joint specimens without fixing imperfections)]. Value one agreed well with the experimental results of single-keyed dry joints in Jiang et al. (2016) [31] as the average and standard deviation are approximately 97.7% and 8.6%, respectively. Value two agreed well with the experimental results of three-keyed dry joints in Jiang et al. (2016) [31] as the average and standard deviation are approximately 109% and 11.1%, respectively.

### 7.2. Modified Formula for Multiple-Keyed Dry Joints Based on AASHTO and This Numeric Study

Therefore, the modified formula is as follow:

(25) $$V_{a}=0.90\times A_{K}\sqrt{6.792\times 10^{-3}f_{cm}}\left(12+2.466\sigma_{n}\right)+0.6A_{sm}\sigma_{n}$$

Compared with the calculated values from AASHTO specification [24] and the modified formula proposed in this study, Table 7 presents the experimental shear capacity of three-keyed dry-joint specimens and single-keyed dry-joint specimens from Jiang et al. (2016) [31] and numeric shear capacity of single-keyed dry-joint specimens in this numeric study. In Jiang et al. (2016) experimental study [31], the shear capacity of single-keyed dry joints was underestimated by AASHTO and by the modified formula 21.1% and 33.5% in average, respectively, similar to those without fixing imperfections in this numeric study (by 18.0% and 30.1%). On the other hand, the shear capacity of three-keyed dry joints in those tests was overestimated by AASHTO 0.888% averagely. At the same time, the modified formula in this study is enough conservative by 9.58% averagely to predict the shear capacity of those three-keyed dry joints. As for the average shear capacity of single-keyed dry-joint specimens with or without fixing imperfections of key in this numeric study, AASHTO and the modified formula underestimates that by 7.32% and 18.4% in average, respectively.

Overall, the modified formula was more appropriately conservative to predict the shear capacity when applied in three-keyed dry joints and single-keyed dry joints with a higher possibility of fixing imperfections. However, the modified formula was over-conservative in the cases of single-keyed dry joints without or with a lower possibility of fixing imperfections, as these joints follow the shearing-off failure mode and have no need to consider the correction factor for direct-shear strength deduced by fixing imperfections.

## 8. Conclusions

With the purpose of better understanding the shear behavior of HSC single-keyed dry joints with fixing imperfections in PCSBs, this study established a FE model validated by an experimental study. The nonlinear behavior of concrete was simulated by using the CDP model in *ABAQUS*, and the numeric results included ultimate shear strength, load–displacement curves, crack patterns and the contour plat of the Maximum principal stress contour. The parametric studies on types of fixing imperfections, confining pressure levels and concrete strengths were investigated by the validated numeric model. The following conclusions could be drawn as below.

(1) Good agreement between experimental data and numeric results was received in this study on single-keyed dry joints without fixing imperfections and validated the numeric model for later parametric studies. The average and standard deviation in the prediction of ultimate shear strength were approximately 98.0% and 7.8%, respectively. Both the simulated load–displacement curves with five stages and the crack patterns were similar to experimental results and AASHTO. This numeric study was better than experimental tests to output the shear loads, load–displacement curves and the crack patterns within the failure.

(2) Compared with single-keyed dry-joint specimens without fixing imperfections of key, those with fixing imperfections were different in shear transfer mechanisms even under the same shear load, which was why the shear capacity was lower, the load–displacement relationship was different with lower initial stiffness and larger vertical ultimate displacement, and cracks propagated in different ways. Less load was transferred from male parts to female parts through the lower surface of the key as the level of maximum principal stress arising at the male key rose, and the regions of stress concentrations increased.

(3) The possibility of fixing imperfections of shear keys should be avoided if possible or considered more carefully, especially fixing imperfections at lower surface of the key. The most unfavorable condition for shear capacity of single-keyed dry joints was the L, reducing the ultimate shear strength by at least 22.8%. The crack propagation mode was similar to the Mode one proposed by Jiang et al. (2015) and crack patterns of three-keyed dry joints from Jiang et al. (2016). The male key was sheared off from left bottom to right top instead of shearing-off failure in the root. Throughout this cracking process, increasing concrete elements attended to sustain shear load so there were at least two peak loads. The last peak load was the ultimate load. R reduced the ultimate shear strength by at least 11.4%. The cracks propagation mode was similar to those from Turmo et al. (2006) and Ahmed et al. (2019), with crack imitating in the female key. The initial stiffness of those with that two fixing imperfections was much lower. The rest of the fixing imperfections made little difference in shear behavior of single-keyed dry joints.

(4) Prestress loss should be avoided if possible or considered more carefully. Ultimate shear strength of all the numeric specimens increased with higher confining pressure. In addition, higher confining pressure (1.0 MPa and 2.0 MPa) alleviated the negative effect of fixing imperfections on shear behavior, including higher initial stiffness, much load transferred, and restrained stress concentrations and cracks propagations in the male part. It was more evident when confining pressure increasing from 0.5 MPa to 1.0 MPa.

(5) When HSC was applied, the possibility of fixing imperfections should be considered more carefully. Higher concrete strength improved the shear behavior of all the numeric specimens, including shear strength, initial stiffness and slightly restrained stress concentrations. Cracks propagated at a lower angle to the horizontal. Less load was transferred from the male part to the female part, but the shear capacity was higher. Higher concrete strength alleviated the negative effect of $MF_{1}$ and $MF_{2}$ on shear capacity of single-keyed dry joints, while the case of L and R was the opposite.

(6) The AASHTO formula was conservative for single-keyed dry joints, but it was not safe enough when applied in three-keyed dry joints and single-keyed dry joints with a higher possibility of fixing imperfections under higher confining pressure. A reduction factor of direct-shear strength, 0.9, was proposed in this numeric analysis study to modify the AASHTO formula for keyed dry joints with a higher possibility of fixing imperfections. The modified formula is enough conservative by 9.58% averagely to predict the shear capacity of three-keyed dry-joint specimens in Jiang et al. (2016) experimental study, and by 7.32% averagely to predict that of single-keyed dry joints with or without fixing imperfections in this numeric study. The modified formula meets the command of the trend of PCSBs.

## Figures and Tables

**Table 1:** Parameter values of stress–strain curves of concrete under uniaxial compression.

$f_{c,r}$ (N/mm^2^)	20	25	30	35	40	45	50	55	60	65	70	75	80
$\varepsilon_{c,r}$ (10^−6^)	1470	1560	1640	1720	1790	1850	1920	1980	2030	2080	2130	2190	2240
$\alpha_{c}$	0.74	1.06	1.36	1.65	1.94	2.21	2.48	2.74	3.00	3.25	3.50	3.75	3.99
$\varepsilon_{cu}/\varepsilon_{c,r}$	3.0	2.6	2.3	2.1	2.0	1.9	1.9	1.8	1.8	1.7	1.7	1.7	1.6

**Table 2:** Parameter values of stress–strain curves of concrete under uniaxial tension.

$f_{t,r}$ (N/mm^2^)	1.0	1.5	2.0	2.5	3.0	3.5	4.0
$\varepsilon_{t,r}$ (10^−6^)	65	81	95	107	118	128	137
$\alpha_{t}$	0.31	0.70	1.25	1.95	2.81	3.82	5.00

**Table 3:** Ultimate shear strength of single-keyed dry joints: experimental [31] versus numeric.

Test Name	$f_{cu}$ (MPa)	Average Vu(E) (kN) (1)	Vu(N) (kN) (2)	$\frac{\left(2\right)}{\left(1\right)}$	Appearance of the First Crack (Male Part; Numeric Value)			Appearance of Shear-off Crack (Numeric Value)		
					Angle (°)	Load (kN) (3)	$\frac{\left(3\right)}{\left(2\right)}$ (%)	Angle (°)	Load (kN) (4)	$\frac{\left(4\right)}{\left(2\right)}$ (%)
MK1-H5-0.5	59.9	87.3	93.9	1.08	62	43.6	46.5	95	89.7	95.5
MK1-H5-1.0	59.9	123	117	0.955	45	58.4	49.9	95	111	94.9
MK1-H5-2.0	59.9	139	129	0.923	27	67.2	52.2	95	125	97.3
MK1-H7-0.5	70.3	105	115	1.09	66	54.0	47.1	95	113	98.3
MK1-H7-1.0	70.3	134	130	0.976	51	65.8	50.4	90	129	99.3
MK1-H7-2.0	70.3	165	141	0.859	48	80.7	57.0	90	140	99.0
Average				0.980	/	/	50.5	/	/	97.4
Standard deviation				0.078	/	/	/	/	/	/

Vu(E)—experimental ultimate shear load; Vu(N)—numeric ultimate shear load.

**Table 4:** Four types of fixing imperfections of keys for parametric study.

Specimen	Fixing Imperfections	$d_{1}^{\prime }$ (mm)	$d_{2}^{\prime }$ (mm)	$x_{1}$ (mm)	$x_{2}$ (mm)	Concrete Strength	Confining Pressure (MPa)
MK1-Ha-m	/	0	0	0	0	C70; C50	0.5; 1.0; 2.0
MF_1_K1-Ha-m	MF_1_	0.5	0	0	0	C70; C50	0.5; 1.0; 2.0
MF_2_K1-Ha-m	MF_2_	0	0.5	0	0	C70; C50	0.5; 1.0; 2.0
RK1-Ha-m	R	0	0	0	0.5	C70; C50	0.5; 1.0; 2.0
LK1-Ha-m	L	0	0	0.5	0	C70; C50	0.5; 1.0; 2.0

**Table 5:** Reduced percentage of numeric value of single-keyed dry joints with fixing imperfections of keys.

Specimen	Numeric Ultimate Strength		AASHTO		Specimen	Numeric Ultimate Strength		AASHTO	
	Value (kN)	Reduced by (%)	Value (kN)	Error (%)		Value (kN)	Reduced by (%)	Value (kN)	Error (%)
MK1-H5-0.5	93.9	/	83.2	−12.8	MK1-H7-0.5	114	/	93.5	−22.8
MF_1_K1-H5-0.5	94.5	−0.682		−13.6	MF_1_K1-H7-0.5	115	−0.331		−23.2
MF_2_K1-H5-0.5	91.7	2.38		−10.1	MF_2_K1-H7-0.5	112	2.10		−20.2
RK1-H5-0.5	73.0	22.2		12.3	RK1-H7-0.5	92.5	19.4		1.05
LK1-H5-0.5	66.0	29.7		20.7	LK1-H7-0.5	74.6	35.0		20.2
MK1-H5-1.0	117	/	93.7	−24.9	MK1-H7-1.0	130	/	105	−24.3
MF_1_K1-H5-1.0	116	0.752		−24.0	MF_1_K1-H7-1.0	131	−0.184		−24.6
MF_2_K1-H5-1.0	119	−1.46		−26.7	MF_2_K1-H7-1.0	138	−5.77		−31.5
RK1-H5-1.0	104	11.5		−10.5	RK1-H7-1.0	108	17.3		−2.84
LK1-H5-1.0	90.4	22.8		3.53	LK1-H7-1.0	95.8	26.6		8.77
MK1-H5-2.0	129	/	115	−12.2	MK1-H7-2.0	141	/	128	−10.7
MF_1_K1-H5-2.0	118	8.05		−3.18	MF_1_K1-H7-2.0	141	0.474		−10.2
MF_2_K1-H5-2.0	121	6.25		−5.20	MF_2_K1-H7-2.0	143	−1.32		−12.2
RK1-H5-2.0	114	11.4		0.602	RK1-H7-2.0	123	12.8		3.45
LK1-H5-2.0	99.1	23.0		13.6	LK1-H7-2.0	99.7	29.5		22.0

Error = (1 − Vu(N)/Vu(AASHTO)) × 100%.

**Table 6:** Direct-shear strength of keyed dry joints: experimental values (from Jiang et al. (2016) [31]) versus numeric values.

Concrete Strength (MPa)	Confining Pressure (MPa)	Direct-Shear Strength of Each Shear Key for Keyed Dry-Joint Specimens				(1)/(3)	(2)/(4)	(2)/(1)
		Numeric Value		Experimental Value				
		(1)	(2)	(3)	(4)			
C50	0.5	90.9	80.8	84.3	70.5	1.08	1.15	0.889
C50	1.0	111	103	116.	83.8	0.957	1.23	0.929
C50	2.0	117	104	127	98.9	0.916	1.05	0.893
C70	0.5	112	98.9	102	88.6	1.09	1.12	0.885
C70	1.0	124	115	128	100	0.975	1.14	0.920
C70	2.0	130	118	153	134	0.848	0.877	0.909
Average/ Standard deviation						0.977/ 0.086	1.09/ 0.111	0.904

Value (1): numeric single-keyed dry-joint specimens without fixing imperfections of key; Value (2): summation of numeric single-keyed dry-joint specimens with or without four fixing imperfections divided by five; Value (3): experimental single-keyed dry-joint specimens; Value (4): direct-shear strength of experimental three-keyed dry-joint specimens divided by three; direct-shear strength of each shear key for keyed dry joints specimens [24]: the ultimate shear loads minus the friction force (a.k.a., “$V_{direct-shear\;strength}=V_{\left(E\;or\;N\right)}-0.6A_{sm}\sigma_{n}$”).

**Table 7:** Comparisons between (experimental [31]) numeric ultimate shear strength and predicted formula.

Specimen	Key Number	Ultimate Shear Strength				Error ^1^ (%)	Error ^2^ (%)	Error ^3^ (%)	Error ^4^ (%)
		(Average) Experimental Value	(Average)Numeric Value	AASHTO (Equation (24))	Modified Formula (Equation (25))				
		(1)	(2)	(3)	(4)				
Experimental specimens from Jiang et al. (2016)									
K3-H5-0.5	3	218	/	247	223	11.8	/	2.24	/
K3-H5-1.0	3	263	/	275	249	4.30	/	−5.82	/
K3-H5-2.0	3	321	/	332	301	3.42	/	−6.45	/
K3-H7-0.5	3	272	/	277	250	2.10	/	−8.51	/
K3-H7-1.0	3	313	/	309	279	−1.30	/	−12.1	/
K3-H7-2.0	3	427	/	371	337	−15.0	/	−26.8	/
Average						0.888	/	−9.58	/
Experimental specimens from Jiang et al. (2016) and numeric specimens without fixing imperfections from this study									
K1-H5-0.5	1	87.3	93.9	83.2	75.2	−4.90	−12.8	−16.1	−24.9
K1-H5-1.0	1	123	117	93.7	84.9	−30.8	−24.9	−44.3	−37.8
K1-H5-2.0	1	139	129	115	104	−21.6	−12.2	−33.6	−23.3
K1-H7-0.5	1	105	115	93.5	84.5	−12.8	−22.8	−24.9	−35.9
K1-H7-1.0	1	134	130	105	95.1	−27.4	−24.3	−40.7	−37.3
K1-H7-2.0	1	165	141	128	116	−28.8	−10.7	−41.7	−21.7
Average						−21.1	−18.0	−33.5	−30.1
Numeric single-keyed dry-joint specimens with or without fixing imperfections from this study									
K1-H5-0.5	1	/	83.8	83.2	75.2	/	−0.721	/	−11.5
K1-H5-1.0	1	/	109	93.7	84.9	/	−16.5	/	−28.6
K1-H5-2.0	1	/	116	115	104	/	−1.29	/	−11.3
K1-H7-0.5	1	/	102	93.5	84.5	/	−8.97	/	−20.7
K1-H7-1.0	1	/	121	105	95.1	/	−14.9	/	−26.9
K1-H7-2.0	1	/	130	128	116	/	−1.52	/	−11.6
Average							−7.32		−18.4

Error ^1^: $\left[1-\frac{\left(1\right)}{\left(3\right)}\right]\times 100\%$; Error ^2^: $\left[1-\frac{\left(2\right)}{\left(3\right)}\right]\times 100\%$; Error ^3^: $\left[1-\frac{\left(1\right)}{\left(4\right)}\right]\times 100\%$; Error ^4^: $\left[1-\frac{\left(2\right)}{\left(4\right)}\right]\times 100\%$.
